# Editorial: Checkpoint inhibition in hematologic malignancies

**DOI:** 10.3389/fonc.2024.1429854

**Published:** 2024-06-03

**Authors:** Jeffrey J. Pu, Joseph J. Drabick, Susan E. Prockop

**Affiliations:** ^1^ VA Boston/Brigham & Women Hospital, Harvard Medical School, Boston, MA, United States; ^2^ Pennsylvania State University Hershey Cancer Institute, Hershey, PA, United States; ^3^ Dana-Farber/Boston Children’s Hospital, Harvard Medical School, Boston, MA, United States

**Keywords:** immune checkpoint inhibiter, hypomethylation agent, hematological malignancy, PD-1/PDL-1, CTLA-4, leukemia, lymphoma, T lymphocytes

After decades of sequential but incremental advancements, the armamentarium of cancer therapies has shifted cytotoxic chemotherapy to therapies targeting cell signaling pathways and immunotherapy (1). The crown jewelry of immunotherapy is “checkpoint inhibition”. Under normal physiologic conditions, immune checkpoints (ICs) act as self-regulatory and self-tolerant mechanisms negatively modulating effector T cells to prevent autoimmunity. Inhibition of ICs i can increase cytotoxicity of effector T cells targeting diverted or abnormal cells (2). The huge success of PD-1/PDL-1 axis and CTLA-4 inhibitors in treating relapsed/refractory cancer in both the clinical trial and real-world practice settings has validated this therapeutic strategy (3, 4). This revolutionized therapy has significantly shifted the treatment landscape for solid tumors especially for renal cancer, lung cancer, and melanoma. However, these therapies have thus far been less effective for hematologic malignancies with only certain types of Hodgkin’s lymphomas demonstrating significant responses to PD-1/PDL-1 inhibitors (5–7). This Research Topic collected 7 publications: 2 original research papers, 2 biostatistical analytic papers (one bibliometric analysis and another one meta-analysis), and 3 systemic reviews. The objective of this Research Topic is to update our readers on the current landscape of investigation of IC inhibitor therapy for hematologic malignancies.

In this Research Topic, Radpour et al. reported a data set of histone methylation status of IC receptors in bone marrow infiltrating CD8+ and CD4+ T cells from 30 patients with acute myeloid leukemia (AML) (*Epigenetic Silencing of IC-Receptors in AML*). To analyze the interactions of IC ligands and receptors in AML, this study performed transcriptomic analysis of FACS-purified leukemia stem/progenitor cells (LSC) and paired bone marrow (BM)-infiltrating CD4+ and CD8+ T cells. The IC receptor gene expression was decreased on BM-infiltrating CD8+ T cells and partially decreased on CD4+ T cells via histone deacetylation caused pathological chromatin remodeling. These data suggest that CD8+ T cell dysfunction could be the reason for suboptimal responses to IC inhibition in patients with AML. These findings raise the possibility that combination of hypomethylation agents may improve the efficacy of IC inhibitor therapy.

Romine et al. explored the relationship between regulatory T (Treg) cells exhaustion and IC inhibition by evaluating *ex vivo* responses to small molecules in a variety of cell populations, and the mutational signatures carried by these populations (*Immune cell proportions correlate with clinicogenomic features and ex vivo drug responses in acute myeloid leukemia*). In this study, 560 patients with AML from the Beat AML dataset were analyzed (8). Their data demonstrated that the proportion of T cells of specific lineage is correlated with certain common AML mutations, especially, low T cell signatures being associated with *de novo* TP53 mutations in AML. This study also showed that immune cell signatures can predict patients’ response to certain small molecules. After screening 152 small molecules as monotherapies, they observed that AML samples with high monocycte scores exhibited resistance to both venetoclax and Palbociclib, and sensitivity to BETi and OTX-015. In AML samples with high proportions of Treg, NVPAEW-541, BMS-754807, and metformin demonstrated the highest inhibition. Th1 cell and MPP cell proportions were correlated to azacitidine sensitivity. Furthermore, this study showed that high-monocyte samples have decreased *ex vivo* responses to IC inhibitor therapy and higher CD8+ cell signatures. This study revealed the heterogenous nature of AML in response to therapy, and the importance of developing precise and personalized therapeutic strategies in managing AML patients.

Gómez-Llobell et al. performed a meta-analysis including 13 studies of IC inhibitor therapy in AML (*Immune Checkpoint Inhibitors in Acute Myeloid Leukemia: A Meta-Analysis*). Overall Response (ORR) rate across these studies was 42% (IC95%, 31% - 54%) and Complete Response/Complete Response with incomplete count recovery (CR/CRi) rates was 33% (IC95%, 22%-45%). Mean overall survival (OS) rate was 8.9 months [median 8 months, (IC95%, 3.9 - 15.5)], average OS in first line was 12.0 months and in refractory/relapse (R/R) patients was 7.3 months. The most specific adverse events (AEs) of these therapies are immune-related adverse events (irAEs), derived from their inflammatory effects. The incidence of Grade ≥3 irAEs was low and similar among studies [12% (95%CI 8% - 16%)]. They biostatistically analyzed data confirmed our clinical observation that the efficacy of current IC inhibition therapies for AML is limited. Further work needs to be done to optimize the therapeutic strategy. A bibliometric analysis of 803 publications on IC inhibitor therapy associated immune-related adverse events (irAEs) summarized global research trends (*Bibliometric analysis of rheumatic immune related adverse events associated with immune checkpoint inhibitors*) (Zeng et al.). This study revealed the rapid global growth of irAEs investigation in the last decade. One paper by Du et al. [*Precise diagnosis and targeted therapy of nodal T-follicular helper cell lymphoma (T-FHCL)*] reviewed current therapeutic options for a rare T- cell lymphoma, nodal T-follicular helper cell lymphoma (T-FHCL), in which PD-1/PDL-1 therapy and hypomethylation therapy mediate responses.

Two review papers in this Research Topic [*Checkpoint inhibition in hematologic malignancies* (Tsumura et al.) and *Immune checkpoint blockade in hematological malignancies: current state and future potential* (Pophali et al.)] systemically describe current development and status of IC inhibitor therapy in hematological malignant disease. The authors at both publications concluded that current therapeutic strategies involving PD-1/PDL-1 and CTLA-4 inhibitors are not successful in managing hematological malignancies, especially R/R diseases, except for Hodgins Lymphomas. Recent efforts have been directed to identifying and targeting novel T-lymphocyte ICs, such as LAG-3, TIM-3, TIGIT, and macrophage checkpoint, CD47 (9–14).

This set of manuscripts supports the premise that ongoing work will result in an optimal approach to IC inhibitor therapy for hematological malignancies.

## Author contributions

JP: Conceptualization, Writing – original draft, Writing – review & editing. JD: Writing – review & editing. SP: Writing – review & editing.
